# Validation of a Malay Version of the Smartphone Addiction Scale among Medical Students in Malaysia

**DOI:** 10.1371/journal.pone.0139337

**Published:** 2015-10-02

**Authors:** Siew Mooi Ching, Anne Yee, Vasudevan Ramachandran, Sazlyna Mohd Sazlly Lim, Wan Aliaa Wan Sulaiman, Yoke Loong Foo, Fan kee Hoo

**Affiliations:** 1 Department of Family Medicine, Faculty of Medicine and Health Sciences, Universiti Putra Malaysia, Serdang, Malaysia; 2 Malaysian Research Institute on Ageing, Universiti Putra Malaysia, Serdang, Malaysia; 3 Department of Psychological Medicine, Faculty of Medicine, Universiti Malaya Centre of Addiction Science, University of Malaya, Kuala Lumpur, Malaysia; 4 Department of Medicine, Faculty of Medicine and Health Sciences, Universiti Putra Malaysia, Serdang, Selangor, Malaysia; University of Ariel, ISRAEL

## Abstract

**Introduction:**

This study was initiated to determine the psychometric properties of the Smart Phone Addiction Scale (SAS) by translating and validating this scale into the Malay language (SAS-M), which is the main language spoken in Malaysia. This study can distinguish smart phone and internet addiction among multi-ethnic Malaysian medical students. In addition, the reliability and validity of the SAS was also demonstrated.

**Materials and Methods:**

A total of 228 participants were selected between August 2014 and September 2014 to complete a set of questionnaires, including the SAS and the modified Kimberly Young Internet addiction test (IAT) in the Malay language.

**Results:**

There were 99 males and 129 females with ages ranging from 19 to 22 years old (21.7±1.1) included in this study. Descriptive and factor analyses, intra-class coefficients, t-tests and correlation analyses were conducted to verify the reliability and validity of the SAS. Bartlett’s test of sphericity was significant (p <0.01), and the Kaiser-Mayer-Olkin measure of sampling adequacy for the SAS-M was 0.92, indicating meritoriously that the factor analysis was appropriate. The internal consistency and concurrent validity of the SAS-M were verified (Cronbach’s alpha = 0.94). All of the subscales of the SAS-M, except for positive anticipation, were significantly related to the Malay version of the IAT.

**Conclusions:**

This study developed the first smart phone addiction scale among medical students. This scale was shown to be reliable and valid in the Malay language.

## Introduction

This is no doubt that the smartphone has provided us with enormous convenience in our daily life, as it has a more advanced computing capability and connectivity than basic feature phones [1]. Usage of smartphone has their own variety of goals and purposes. A wide range of studies reported that smartphone has numerous benefits for social and medical purposes [2–5]. Although the smartphone has become one of the most popular and important communication tools, its excessive use has emerged as a social issue worldwide and created a new mental health concern, wherein the user tends to develop dependency on it [6–8].

Smartphone addiction is also called “mobile phone dependence”, “compulsive mobile phone overuse” or “mobile phone overuse”. These terms mainly describe the phenomenon of problematic mobile phone use [9, 10]. “Smartphone addiction” is the term typically used in the literature. This addiction is mainly characterized by excessive or poorly controlled preoccupations, urges, or behaviors regarding smartphone use, to the extent that individuals neglect other areas of life [11–13]. Studies report that excessive mobile phone usage was associated with stress, sleep disturbance, smoking and symptoms of depression [14–16].

Recent data from Malaysia showed that smartphone penetration increased from 47% in 2012 to 63% in 2013. In 2014, 10.13 million Malaysians were active smartphone users, compared with 7.7 million in 2012 [17–20]. Pathological use of the smartphone is similar to internet addiction. Usage of internet addiction becomes excessive among youths and adults worldwide [21]. Excessive internet addiction leads to psychiatric disorders, low self-esteem, depression and impaired academic and occupational performance [22–25]. Local studies reported that the prevalence of internet addiction was 43% [26], and there are more than 4.2 million active Facebook users in Malaysia; in fact, Facebook is the top networking site in this country. Given that there has been a rapid increase in smartphone usage in Malaysia, there is an urgent need to validate a scale to measure smartphone addiction in the local population to determine its prevalence and to identify who is at risk of developing smartphone addiction so that policy makers can plan a suitable intervention in the near future.

Like the factorial structure prepared for internet addiction test [27], the Smartphone Addiction Scale (SAS) developed by Min Kwon et al. was the first scale for smartphone addiction used for diagnosis [28]. This scale consists of 33 items and has been reported to be reliable, with good internal consistency (Cronbach’s alpha = 0.967), and the concurrent validity of the six subscales ranges from 0.32 to 0.61 [28].

This study aimed to translate the SAS into the Malay language and to study the psychometric properties of the Malay version of the SAS (SAS-M) to facilitate its use for further research in the local setting.

## Methodology

### Study Design and Setting

This was a cross-sectional study of all first- and second-year medical students from Universiti Putra Malaysia. These students were approached for a validation study from August 2014 to September 2014. This university is located in Serdang, next to Malaysia’s administrative capital city, Putrajaya. We estimated the sample size to be at least 165 based on the calculation of five cases per item in the SAS (which has a total of 33 items) [29]. Therefore, a sample size of 228 in this study was adequate.

#### Procedure

*Stage 1*: The author obtained the English version of the SAS from Kwon et al. Translation from English to Malay was performed in parallel by two bilingual language experts, and a back translation was performed by a third bilingual language expert. Discrepancies between the original version and the back translation were discussed, and adjustments were made accordingly. A final version of the translated SAS, which we called a draft of the SAS-M, was generated by an expert panel composed of one psychiatrist, two senior physicians and one family physician, all of whom were qualified professionals regarding use of psychometric instruments and all of whom had clinical experience with depressive conditions.

*Stage 2*: The first draft of the SAS-M was pilot tested among 20 native Malay-speaking students to identify any flaws in this version. Any words that the respondents considered unsuitable or inappropriate in this version were noted and corrected. Most students had difficulty in accepting item 15: “Being angry and resentful when I do not have a smartphone”. This item was revised and translated to “Feeling impatient and restless when I do not have a smartphone” in the Malay language. The finalized version of the SAS-M was further reviewed by two consultant psychiatrists with more than 10 years of experience to assess the content validity and to ensure a satisfactory face and satisfactory semantics, criteria, and conceptual equivalence.

*Stage 3*: Each student provided written informed consent after receiving a full explanation of the nature and confidentiality of the study, and 228 students consented to participate in the study, with a non-response rate of 9%. Sociodemographic data (age, gender, ethnicity and household income) were obtained from the students. Information about the students’ smartphone use based on their own estimation, such as the number of hours of use per week, the number of years as a regular smartphone user and the age at which they started to use a smartphone, were documented. The students were given the following questionnaires:

1. The SAS and SAS-M (Table A in S1 Text).
2. Malay version of the Internet Addiction Test.

### Instruments

#### Smartphone Addiction Scale [28]

The SAS is a self-completed, 6-point Likert-type scale with 33 items. Each question has a response scale from 1 to 6 (1 = strongly disagree to 6 = strongly agree), reflecting the frequency of the symptoms. The respondent circles the statement that most closely describes their smartphone use characteristics. The total score possible on the SAS ranges from 48 to 288. The higher the score is, the greater the degree of pathological use of the smartphone.

#### Internet Addiction Test [26]

The IAT questionnaire, which was developed by Kimberly Young in 1998, is the tool most commonly used in diagnosing internet addiction. The Malay version has been validated locally, with good internal consistency (Cronbach’s alpha = 0.91) and parallel reliability (intraclass correlation coefficient (ICC) = 0.88, *P* < 0.001). This is a self-completed questionnaire consisting of a 5-point Likert-type scale containing 20 items, with a minimum point value of 20 and a maximum point of value 100. The scoring of each question ranges from 1 to 5 (1 = never to 5 = always), replicating the occurrence of the symptoms. The students chose the statement that best described the features of their internet use. The higher the score is, the greater the degree of pathological use of the internet is. When the score on the Malay version of the IAT is more than 43, then the individual is diagnosed as at risk of internet addiction [26].

### Statistical Analysis

All analyses were conducted using the Statistical Package for the Social Sciences version 21.0 (SPSS, Chicago, IL, USA). Descriptive statistics were computed for the baseline characteristics of the participants. Cronbach’s alpha was used to assess the internal consistency of the SAS-M, and the normality of the data was assessed using Kolmogorov-Smirnov analysis. The homogeneity of the scale items was analyzed based on correlation coefficients between items and total scores if an item was deleted. Construct validity was investigated by exploratory factor analysis and oblique promax with Kaiser Normalization. Factor loading of > 0.30 was used to determine the items for each factor. Based on Guttman-Kaiser rule, the factors with eigenvalue large than 1 are retained [30, 31]. The ICC was used to examine the parallel reliability between the SAS-M and the English version of the SAS and the test-retest reliability of the SAS-M. Pearson’s correlation was used to examine the concurrent validity between the SAS-M and the Malay version of the IAT. The optimal SAS-M cut-off score for at-risk cases was determined from the coordinate points when the score for the Malay version of the IAT was more than 43 [26], at which point the sensitivity and specificity were optimal in receiver operating characteristic (ROC) analyses. The area under the curve (AUC) was determined for the ROC curve.

### Definition

Regular user is defined as those who use smartphone at least 6 or more times in 6 months [32]

### Ethical Approval

Ethics approval for this study was obtained from the Ethic Committee of the Universiti Putra Malaysia (FPSK-EXP14 P091).

## Results

A total of 228 students were recruited in this study. Table 1 shows the clinical characteristics of the studied population. Overall, the mean age was approximately 22 years ± 1.1. More than half of the students were female (56.6%), and the majority were of Malay ethnicity (52.4%). The mean hours of smartphone use per week was 36.5 hours. On average, the students started using a smartphone at the age of 19 years, and the mean number of years of regular smartphone use was 2.4 years.

**Table 1:** Characteristics of the study population (N = 228).

Variable	Mean(SD)	N (%)
Age (years)	21.7(1.1)	
Gender		
Male		99(43.4)
Female		129(56.6)
Race		
Malay		112(49.1)
Chinese		94(41.2)
Indians		18(7.9)
Other		4(1.8)
Average monthly family income (RM)		
< RM1000		23(10.1)
RM1000-1999		41(18.0)
RM2000-2999		35(15.4)
RM3000-3999		37(16.2)
> RM4000		92(40.4)
Average hours of use per week	36.5(2.4)	
Age at first use of smart phone	18.7(2.5)	
Years of regular smart phone use	2.4(2.3)	

RM- Ringgit Malaysia. The median household income based on the statistic in Malaysia was RM 3,626.

### Factor Structure and Internal Consistency of the SAS-M

Bartlett’s test of sphericity was significant (p < 0.01), and the Kaiser-Meyer-Olkin measure of sampling adequacy for the SAS-M was 0.92, indicating that the scale was meritorious [33], which in turn indicated that the factor analysis was appropriate. Six factors were extracted (eigenvalue >1.00) via the exploratory factor analysis approach and the oblique promax rotation with Kaiser normalization which accounted for 65.3% of the total variance. This result was consistent with the original SAS [28].

The SAS-M exhibited good internal consistency; Cronbach’s alpha coefficient for the total scale was 0.94, and the respective coefficients for the six factors were 0.877, 0.843, 0.865, 0.837, 0.865 and 0.861. The six factors corresponding to the SAS subscales were referred to as “cyberspace-oriented relationship”, “daily life disturbance”, “primacy”, “overuse”, “positive anticipation” and “withdrawal” (Table 2). All items had corrected-item total correlations of more than 0.9. Deletion of any of the items did not increase the internal consistency of the total score (Table 3). The parallel reliability between the SAS-M and the SAS was high, as demonstrated by an ICC of 0.95 (95% *Confidence interval =* 0.937–0.962). The test-retest reliability of the SAS-M after a 1-week interval was high, with an ICC of 0.85 (95% *Confidence interval =* 0.808–0.866).

**Table 2:** Factor analysis of the SAS-Malay version.

Question No:	Components					
	Cyberspace-oriented relationship	Daily life disturbance	Primacy	Overuse	Positive anticipation	Withdrawal
B19	0.694					
B20	0.625					
B21	0.736					
B22	0.738					
B23	0.770					
B24	0.445					
B26	0.694					
B1		0.767				
B2		0.751				
B3		0.636				
B4		0.640				
B5		0.721				
B33		0.415				
B10			0.645			
B11			0.783			
B12			0.684			
B13			0.635			
B14			0.738			
B25				0.566		
B27				0.563		
B28				0.729		
B29				0.677		
B30				0.524		
B31				0.550		
B32				0.564		
B6					0.815	
B7					0.858	
B8					0.897	
B9					0.780	
B15						0.588
B16						0.749
B17						0.726
B18						0.546
Eigenvalue	11.62	3.244	2.339	1.702	1.468	1.163
Variance (%)	35.211	9.829	7.088	5.157	4.447	3.524

Extraction Method: Confirmatory exploratory analysis.

Rotation Method: Oblique promax with Kaiser Normalization.

**Table 3:** Corrected item–Total correlations and Cronbach’s alpha if item was deleted for the SAS-M.

Item	Scale Mean if Item Deleted	Scale Variance if Item Deleted	Corrected Item-Total Correlation	Cronbach’s Alpha if Item Deleted
B1	93.83	536.826	0.471	0.938
B2	93.94	535.771	0.541	0.937
B3	94.15	536.772	0.492	0.938
B4	94.13	533.887	0.530	0.938
B5	94.04	531.368	0.565	0.937
B6	93.05	550.295	0.288	0.940
B7	92.85	545.415	0.399	0.939
B8	92.95	548.266	0.318	0.939
B9	92.93	545.818	0.347	0.939
B10	94.20	528.219	0.633	0.937
B11	94.06	524.956	0.636	0.936
B12	93.48	535.600	0.525	0.938
B13	93.82	528.705	0.625	0.937
B14	93.93	531.469	0.562	0.937
B15	94.20	527.858	0.653	0.936
B16	94.34	530.282	0.657	0.936
B17	94.05	529.584	0.629	0.937
B18	94.43	532.038	0.648	0.936
B19	94.67	538.962	0.465	0.938
B20	94.30	534.322	0.583	0.937
B21	94.26	526.914	0.636	0.936
B22	94.47	529.575	0.642	0.936
B23	94.71	533.587	0.646	0.937
B24	93.88	528.861	0.574	0.937
B25	93.73	532.767	0.514	0.938
B26	94.46	533.527	0.585	0.937
B27	93.09	542.530	0.408	0.939
B28	93.30	533.400	0.449	0.939
B29	93.36	528.506	0.617	0.937
B30	93.97	531.754	0.646	0.937
B31	94.12	532.695	0.604	0.937
B32	93.72	530.699	0.568	0.937
B33	94.32	529.818	0.626	0.937

### Concurrent Validity of the SAS-M: Correlations between the Subscales of the SAS-M and the Malay Version of the IAT

The results of the Pearson correlation analysis that was conducted between the subscales of the SAS-M and the Malay version of the IAT are shown in Table 4. The results show that all of the subscales of the SAS-M, except for “positive anticipation”, were significantly related to the Malay version of the IAT.

**Table 4:** Concurrent validity of SAS-M (Pearson’s correlation): Subscales of the SAS-M and the Malay version of the IAT.

	Cyberspace oriented relationship	Daily life disturbance	Primacy	Over use	Positive anticipation	Withdrawal	SAS-M
**Cyberspace-oriented relationship**	-						
**Daily life disturbance**	0.564^**^	-					
**Primacy**	0.588^**^	0.445^**^	-				
**Overuse**	0.544^**^	0.602^**^	0.50^**^	-			
**Positive anticipation**	0.170^**^	0.160^*^	0.347^**^	0.285^**^	-		
**Withdrawal**	0.658^**^	0.541^**^	0.644^**^	0.561^**^	0.224^**^	-	
**SAS-M**	0.818^**^	0.766^**^	0.783^**^	0.810^**^	0.440^**^	0.803^**^	-
**Malay version of the IAT**	0.583^**^	0.598^**^	0.416^**^	0.573^**^	0.075	0.523^**^	0.645^**^

* = *p* < 0.05

** = *p* < 0.01

The AUC for the ROC curve was 0.801 (95% CI = 0.746 to 0.855). The optimal cut-off score for identifying at-risk cases was more than 98, with a sensitivity of 71.43%, a specificity of 71.03%, a positive predictive value (PPV) of 64.10% and a negative predictive value (NPV) of 77.44%. The prevalence of an at-risk case developing smartphone addiction in this study was 46.9%, based on a score of 98.

## Discussion

This study examined the internal consistency, dimensionality, and concurrent and construct validity of the SAS-M. Findings from the study indicate that the SAS-M is a reliable and valid instrument for assessing smartphone addiction in the Malay-speaking population.

In this study, the SAS-M exhibited good internal consistency; Cronbach’s alpha coefficient for the total scale was 0.94, and the respective coefficients for the six factors were 0.877, 0.843, 0.865, 0.837, 0.865 and 0.861. The parallel reliability of the SAS-M and the test-retest reliability after a 1-week interval were found to be good, with ICCs of 0.95 and 0.85, respectively, which are even better than those of the original version of the SAS [28]. To date, this is the first study of its kind related to smartphone addiction, and it shows that the SAS-M is as good as the English version.

However, the six dominant components that explained a large proportion of the variability of the SAS-M were similar to those of the original SAS. In the present study, the components consisted of “cyberspace-oriented relationship”, “daily life disturbance”, “primacy”, “overuse”, “positive anticipation” and “withdrawal”. The components in the original SAS were “daily life disturbance”, “positive anticipation”, “withdrawal”, “cyberspace-oriented relationship”, “overuse” and “tolerance”. Not all of the factors acquired in this factor analysis paralleled to the factors obtained in the original SAS. It is more likely due to the fact that this reflects the differences between Malay and Korean samples. The meaning of the original SAS had been changed during the translation process.

The majority of the components reported in the current study are the same, except for the component “primacy”, which is different from the component “tolerance” in the original SAS. The possible reasons could be our study population were younger (21.7 ± 1.1 years with an age range from 20 to 27) compared to the Korean population (26.1± 6.0 with age ranges from 18 to 53). The background our study population was homogenous as all the subjects were medical students compared to the wide range of the occupation and education level in the original SAS study. The different interpretation could be complicated by the heterogeneity in the backgrounds and education of the studied population.

In this study, all of the subscales of the SAS-M, except for “positive anticipation”, were significantly related to the Malay version of the IAT. This may be the only subscale that does not correlate well with the IAT because the IAT mainly measures adverse use of the internet, so there are no items asking about positive anticipation. Nevertheless, this aspect does not reduce the concurrent validity because the other 5 subscales are strongly correlated.

The prevalence of at-risk cases that could be identified as smartphone addiction using this scale was 46.9%. There are several possible explanations for this result. The high prevalence of smartphone addiction is expected as a local study has shown that 85% of Malaysians own mobile phones [18]. Smartphones are the favorite option because Malaysians tend to follow trends in the community [20]. In addition, smartphone provides free instant messaging through certain platforms, e.g., WhatsApp and WeChat, which enrich the lives of users. Entertainment is another possible explanation of the high prevalence of smartphone addiction because with these phones, medical students can listen to music, watch movies and play games to relieve stress [34]. Therefore, they may tend to spend more time with their smartphone at the end of the day and to ultimately become pathological users.

However, one of the concern in our study would be the optimal SAS-M cut-off score for at-risk cases was determined from the coordinate points when the score for the Malay version of the IAT was more than 43. This is not up to date well-established cut-offs for IAT. Similarly there is no established diagnostic criterion of internet or smartphone addiction according to DSM V in the spectrum of addiction disorder [21, 25]. Thus, the cut-off point proposed by our study was probably too low leading to a very high estimated rate of smartphone addiction. By right the diagnosis of internet addiction should be based on three criteria as described by Ko, et al, 2012 [25].

SAS-M functions more like a screening or a scale for the assessment of the severity of addictive use of smartphone than a diagnostic instrument. Making a proper diagnosis of smartphone addiction will be an important issue for future research. We proposed that in the future the diagnosis of smartphone addiction should include more criteria which consist of criteria A, B, and C. Criterion A contains six characteristic symptoms of smartphone addiction like cyberspace orientated relationship, daily life disturbance, primacy, overuse, positive anticipation and withdrawal. Criterion B needs to include the functional impairment secondary to smartphone use. Criterion C should exclude other psychiatric disorder like bipolar disorder or other impulsive disorder. Subjects who fulfilling all the criteria A, B, and C would only be considered to have smartphone addiction.

### Strength and limitations

The results of this study should be interpreted in the context of the study's limitations: First, there is no established diagnostic criterion for internet or smartphone addiction according to DSM V in the spectrum of addiction disorder [21, 25]. However, in view of the limited studies in smartphone addiction in local setting, the results of this study still can give some insights to the health care professional team. Second, despite the sample size was adequate but it was not randomized. The gender and the race were not equally distributed. Moreover, this study was conducted at a single center, so the sample population was homogenous and may not reflect the general population of Malaysia.

Despite this limitation, the results of the present study proven that the SAS-M can be used for the evaluation of smartphone addiction among educated Malaysian young adults.

## Conclusion

This study developed the first smart phone addiction scale among medical students. This study also provides evidence that the SAS-M is a valid and reliable, self-administered tool to screen for those at risk of having smartphone addiction.

## Supporting Information

S1 Text Smart phone addiction Malay Version Questionnaire. (DOC)
