# Supplementary material for: Validation of a Malay Version of the Smartphone Addiction Scale among Medical Students in Malaysia
Source: PLoS One. 2015 Oct 2;10(10):e0139337. doi: 10.1371/journal.pone.0139337 (PMC4592235; doi:10.1371/journal.pone.0139337)
Supplement: S1 Text — (DOC) [file pone.0139337.s001.doc]

**S1 Table: Smart phone addiction Malay Version Questionnaire**

| **Butiran / Items** | | **Sangat tidak setuju /Strongly disagree** | **Tidak setuju/ Disagree** | **Agak tidak setuju / Weakly disagree** | **Agak setuju/ Weakly agree** | **Setuju/ Agree** | **Sangat setuju/ Strongly agree** |
| --- | --- | --- | --- | --- | --- | --- | --- |
| 1 | Kerja yang dirancang tidak dapat dilakukan akibat penggunaan telefon pintar | 1 | 2 | 3 | 4 | 5 | 6 |
| 2 | Sukar memberi tumpuan dalam kelas, semasa membuat tugasan, atau semasa bekerja akibat penggunaan telefon pintar | 1 | 2 | 3 | 4 | 5 | 6 |
| 3 | Mengalami pening kepala atau penglihatan kabur akibat penggunaan telefon pintar yang berlebihan | 1 | 2 | 3 | 4 | 5 | 6 |
| 4 | Rasa sakit di pergelangan tangan atau di tengkuk semasa menggunakan telefon pintar | 1 | 2 | 3 | 4 | 5 | 6 |
| 5 | Berasa letih dan kurang tidur akibat penggunaan telefon pintar yang berlebihan | 1 | 2 | 3 | 4 | 5 | 6 |
| 6 | Berasa tenang dan selesa semasa menggunakan telefon pintar | 1 | 2 | 3 | 4 | 5 | 6 |
| 7 | Berasa seronok dan teruja semasa menggunakan telefon pintar | 1 | 2 | 3 | 4 | 5 | 6 |
| 8 | Berasa yakin semasa menggunakan telefon pintar | 1 | 2 | 3 | 4 | 5 | 6 |
| 9 | Mampu menghilangkan stres dengan telefon pintar | 1 | 2 | 3 | 4 | 5 | 6 |
| 10 | Tidak ada perkara yang lain yang lebih menyeronokkan daripada menggunakan telefon pintar | 1 | 2 | 3 | 4 | 5 | 6 |
| 11 | Hidup saya kosong tanpa telefon pintar | 1 | 2 | 3 | 4 | 5 | 6 |
| 12 | Berasa sangat bebas semasa menggunakan telefon pintar | 1 | 2 | 3 | 4 | 5 | 6 |
| 13 | Menggunakan telefon pintar adalah perkara yang paling menyeronokkan | 1 | 2 | 3 | 4 | 5 | 6 |
| 14 | Tidak mampu bertahan tanpa telefon pintar | 1 | 2 | 3 | 4 | 5 | 6 |
| 15 | Berasa tidak sabar dan gelisah apabila saya tidak memegang telefon pintar | 1 | 2 | 3 | 4 | 5 | 6 |
| 16 | Sentiasa terfikir tentang telefon pintar saya walaupun semasa saya tidak menggunakannya | 1 | 2 | 3 | 4 | 5 | 6 |
| 17 | Saya tidak akan berhenti daripada menggunakan telefon pintar walaupun kehidupan harian saya sangat terganggu olehnya | 1 | 2 | 3 | 4 | 5 | 6 |
| 18 | Berasa geram apabila saya diganggu semasa menggunakan telefon pintar saya | 1 | 2 | 3 | 4 | 5 | 6 |
| 19 | Membawa telefon ke tandas walaupun saya dalam keadaan tergesa-gesa untuk ke situ | 1 | 2 | 3 | 4 | 5 | 6 |
| 20 | Berasa hebat berjumpa lebih ramai orang melalui penggunaan telefon pintar | 1 | 2 | 3 | 4 | 5 | 6 |
| 21 | Berasa hubungan dengan rakan-rakan di telefon pintar lebih rapat daripada hubungan dengan kawan-kawan sebenar | 1 | 2 | 3 | 4 | 5 | 6 |
| 22 | Terasa perit seperti kehilangan rakan jika tidak dapat meggunakan telefon pintar | 1 | 2 | 3 | 4 | 5 | 6 |
| 23 | Berasa rakan di telefon pintar lebih memahami saya berbanding rakan sebenar | 1 | 2 | 3 | 4 | 5 | 6 |
| 24 | Sentiasa memeriksa telefon pintar supaya tidak terlepas perbualan di kalangan orang-orang lain di Twitter atau Facebook | 1 | 2 | 3 | 4 | 5 | 6 |
| 25 | Memeriksa PJS (Perkhidmatan Jaringan Sosial) seperti Twitter atau Facebook sebaik saja bangun daripada tidur | 1 | 2 | 3 | 4 | 5 | 6 |
| 26 | Memilih untuk bercakap dengan rakan-rakan di telefon pintar daripada secara bersemuka dengan rakan atau anggota keluarga yang lain | 1 | 2 | 3 | 4 | 5 | 6 |
| 27 | Lebih suka mencari informasi menggunakan telefon pintar daripada bertanya kepada orang lain | 1 | 2 | 3 | 4 | 5 | 6 |
| 28 | Bateri telefon pintar yang telah dicaj penuh tidak dapat bertahan sehari | 1 | 2 | 3 | 4 | 5 | 6 |
| 29 | Menggunakan telefon pintar lebih lama daripada yang saya jangkakan | 1 | 2 | 3 | 4 | 5 | 6 |
| 30 | Berasa ingin menggunakan telefon pintar sebaik saja saya berhenti menggunakannya | 1 | 2 | 3 | 4 | 5 | 6 |
| 31 | Telah cuba beberapa kali untuk mengurangkan masa menggunakan telefon pintar, tetapi selalu gagal | 1 | 2 | 3 | 4 | 5 | 6 |
| 32 | Saya selalu berfikir untuk mengurangkan masa dalam penggunaan telefon pintar saya | 1 | 2 | 3 | 4 | 5 | 6 |
| 33 | Orang-orang di sekeliling mengatakan penggunaan telefon pintar saya adalah terlalu kerap | 1 | 2 | 3 | 4 | 5 | 6 |
